# Novel dual multiplex real-time RT-PCR assays for the rapid detection of SARS-CoV-2, influenza A/B, and respiratory syncytial virus using the BD MAX open system

**DOI:** 10.1080/22221751.2021.1873073

**Published:** 2021-01-19

**Authors:** Hsing-Yi Chung, Ming-Jr Jian, Chih-Kai Chang, Jung-Chung Lin, Kuo-Ming Yeh, Chien-Wen Chen, Sheng-Kang Chiu, Yi-Hui Wang, Shu-Jung Liao, Shih-Yi Li, Shan-Shan Hsieh, Shih-Hung Tsai, Cherng-Lih Perng, Ji-Rong Yang, Ming-Tsan Liu, Feng-Yee Chang, Hung-Sheng Shang

**Affiliations:** a Division of Clinical Pathology, Department of Pathology, Tri-Service General Hospital, National Defense Medical Center, Taipei, Taiwan, ROC; b Division of Infectious Diseases and Tropical Medicine, Department of Medicine, Tri-Service General Hospital, National Defense Medical Center, Taipei, Taiwan, ROC; c Division of Pulmonary and Critical Care Medicine, Department of Medicine, Tri-Service General Hospital, National Defense Medical Center, Taipei, Taiwan, ROC; d Department of Emergency Medicine, Tri-Service General Hospital, National Defense Medical Center, Taipei, Taiwan, ROC; e Centers for Disease Control, Taipei, Taiwan, ROC

**Keywords:** SARS-CoV-2, COVID-19, influenza, respiratory syncytial virus, simultaneous detection, real-time PCR, BD MAX platform, Taiwan

## Abstract

SARS-CoV-2 has spread rapidly, causing deaths worldwide. In this study, we evaluated the performance of the BD MAX Open System module for identifying viral pathogens, including SARS-CoV-2, in nasopharyngeal specimens from individuals with symptoms of upper respiratory tract infection. We developed and validated a rapid total nucleic acid extraction method based on real-time reverse transcription-polymerase chain reaction (RT-PCR) for the reliable, high-throughput simultaneous detection of common cold viral pathogens using the BD MAX Platform. The system was evaluated using 205 nasopharyngeal swab clinical samples. For assessment of the limit of detection (LoD), we used SARS-CoV-2, influenza A/B, and respiratory syncytial virus (RSV) RNA standards. The BD MAX dual multiplex real-time RT-PCR panel demonstrated a sensitivity comparable to that of the World Health Organization-recommended SARS-CoV-2 assay with an LoD of 50 copies/PCR. The LoD of influenza A/B and RSV was 100–200 copies/PCR. The overall percent agreement between the BD MAX panel and laboratory-developed RT-PCR test on 55 SARS-CoV-2-positive clinical samples was 100%. Among the 55 positive cases of COVID-19 analysed, no coinfection was detected. The BD MAX rapid multiplex PCR provides a highly sensitive, robust, and accurate assay for the rapid detection of SARS-CoV-2, influenza A/B, and RSV.

## Introduction

In 2019, a cluster of pneumonia cases of unknown aetiology was reported in Wuhan, Hubei Province, China [1–4]. Subsequently, the world witnessed the emergence of an outbreak of severe acute respiratory syndrome-coronavirus 2 (SARS-CoV-2) in the human population, causing COVID-19, which rapidly spread across the globe [5–8]. On 11 March 2020, the COVID-19 outbreak was characterized as a pandemic by the World Health Organization (WHO) [9,10]. Measures taken to reduce its spread critically depend on the timely and accurate identification of individuals suspected of infection. Currently, real-time reverse transcription-polymerase chain reaction (RT-PCR) is considered the gold standard in the detection of SARS-CoV-2, because of its high sensitivity [11–14]. In our previous study, we developed a multiplex PCR test for detecting the *E* and *RdRp* genes of SARS-CoV-2 directly from clinical samples using the open system mode of the BD MAX instrument [15]. However, Lu et al*.* [16] reported that influenza A virus increased the diagnostic difficulties in SARS-CoV-2-infected patients. In addition, several recent case reports have suggested that concurrent infections of SARS-CoV-2 with other pathogens such as influenza virus and other seasonal coronaviruses might influence the morbidity and mortality of patients with COVID-19 [16–20]. Lansbury et al*.* [21] reported that viral coinfections were in the range of 3% among people infected with SARS-CoV-2. More specifically, coinfections with respiratory syncytial virus (RSV) and influenza A accounted for the majority of these viral coinfection cases. Therefore, the presence or absence of a coinfection in COVID-19 cases would rely on correct and rapid identification, which could facilitate the effective confrontation of the virus and reduce patient mortality.

Currently, clinicians cannot rule out a SARS-CoV-2 infection by ruling in other respiratory pathogens at this stage of the COVID-19 pandemic [18]. Therefore, it is crucial to detect the clinical aetiology to accurately rule out SARS-CoV-2 or other upper respiratory viral infections and to appropriately monitor coinfections in patients with COVID-19. In this study, we developed a dual multiplex PCR analysis system to simultaneously detect SARS-CoV-2, influenza A/B, and RSV on the BD MAX platform. To our knowledge, this is the first study proposing such a method to simultaneously detect these viruses in a single assay. We evaluated the clinical performance and analytical sensitivity of this dual multiplex PCR on the BD MAX system. We also retrospectively investigated the presence of influenza A/B and RSV to identify the infection and coinfection status among the 205 nasopharyngeal swab specimens tested in our study.

## Materials and methods

### Study design and clinical specimens

This study was registered on 20 March 2020 and was approved by the Tri-Service General Hospital Institutional Review Board (TSGH IRB C202005041). According to the recommendations from the Taiwan Centers for Disease Control (CDC) and WHO guidelines, we targeted the *E* and *RdRp* genes of SARS-CoV-2 as screening and confirmatory assays. According to the protocol suggested by the Taiwan CDC, we employed one-step real-time RT-PCR using the primer and probe sequences reported by Corman et al. [22].

The experimental procedure and interpretation of results have been previously described [15,23]. In brief, we performed *E* and *RdRp* gene targeting as screening and confirmatory assays by the recommendations from Taiwan CDC. We detected the SARS-CoV-2 *N* gene as an additional confirmatory assay in cases of a positive or inconclusive result. All positive samples were further confirmed by the Taiwan CDC central laboratory. We included 205 residual nasopharyngeal swab specimens collected (COPAN COVID-19 Collection & Transport Kits with Universal Transport Medium or Virus Transport Swabs 147C) from patients suspected of COVID-19 from February 2020 to August 2020. Of the 205 patient specimens, 104 samples were from males and 101 samples were from females, ranging in age from 13 to 98 years. The average age was 61 years. The most common symptoms at illness onset were fever (201, 98.0%), cough (111, 54.1%), and difficulty breathing (69, 33.7%).

### Dual multiplex PCR procedure on the BD MAX system

**Figure 1.**
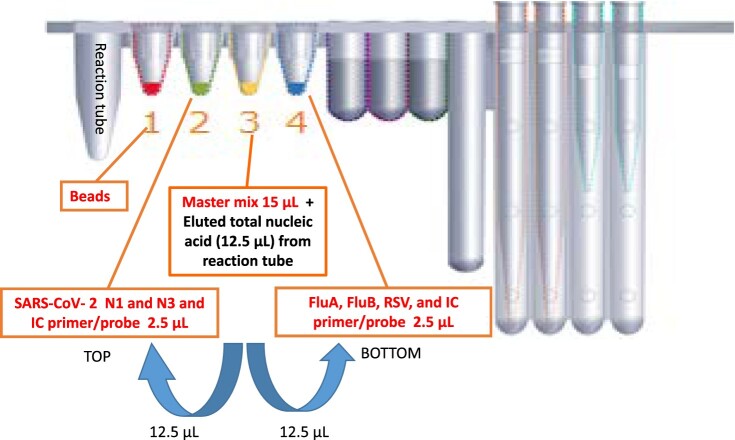
Experimental design for detecting SARS-CoV-2, influenza A/B and respiratory syncytial virus on the BD MAX System.

The dual multiplex PCR test was performed on the BD MAX System, using the BD MAX ExK TNA-3 extraction kit (BD Diagnostic Systems, Québec, QC, Canada) speciﬁc primers and probes designed to detect SARS-CoV-2, influenza A/B, and RSV (Table 1) [24–26]. Briefly, 300 μL of a specimen was aliquoted into the sample buffer tube (SBT) provided with the BD MAX ExK TNA-3 kit and placed in the BD MAX System. Subsequently, 15 μL of 2X SensiFAST Probe No-ROX One-Step mix, 0.3 μL of reverse transcriptase, and 0.6 μL of RiboSafeRNase inhibitor were added to position 3 on the BD cartridge (Figure 1). A 2.5-μL mixture of primers (2.4 pmol/μL) and probe (1.2 pmol/μL) of SARS-CoV-2, internal control target gene, and influenza A/B, RSV, and internal control genes was added to positions 2 and 4. Finally, 12.5 μL eluted nucleic acid was added to position 3 of the cartridge and mixed with the RT-PCR master mixture. A total of 12.5 μL of the above mixture was added to position 2 and mixed with the preloaded 2.5-μL sample. A residual 12.5-μL mixture was added to position 4. The mixture in positions 2 and 4 was transferred to the TOP/BOTTOM PCR chamber (Figure 1). The entire sample-to-result procedure was performed in the BD MAX PCR Cartridges. The cycling condition for SARS-CoV-2 detection was as follows: 50°C for 10 min; 95°C for 2 min; and 45 cycles at 95°C for 10 s, 68°C for 10 s, and 72°C for 13 s for the detection of SARS-CoV-2. The cycling condition for influenza A/B and RSV detection was 50°C for 10 min; 95°C for 2 min; and 45 cycles at 95°C for 10 s, 55°C for 25 s, and 64°C for 32 s.

**Table 1.** Primer and probe sequences used for real-time RT-PCR detection of SARS-CoV-2*, influenza A/B, and RSV*.

Target	Target gene	Primer name	Sequence (5′→3′)	Reference
SARS-CoV-2	*N1*	2019- nCoV_N1-F	GACCCCAAAATCAGCGAAAT	[24]
		2019- nCoV_N1-R	TCTGGTTACTGCCAGTTGAATCTG	
		2019- nCoV_N1-P	/56-FAM/ACCCCGCAT/ZEN/ TACGTTTGGTGGACC/3IABkFQ/BBQ	
	*N3*	2019- nCoV_N3-F	GGGAGCCTTGAATACACCAAAA	
		2019- nCoV_N3-R	TGTAGCACGATTGCAGCATTG	
		2019- nCoV_N3-P	/56-TAMN/AYCACATTGGCACCCGCAATCCTG /3IAbRQSp/	
Human RNase	P	RP-F	AGATTTGGACCTGCGAGCG	
		RP-R	GAGCGGCTGTCTCCACAAGT	
		RP-P	Cy5.5-TTCTGACCTGAAGGCTCTGCGCG-BHQ-3	
Influenza A	*M*	InfA-F	CCMAGGTCGAAACGTAYGTTCTCTCTATC	[25]
		InfA-R	TGACAGRATYGGTCTTGTCTTTAGCCAYTCCA	
		InfA-P	HEX-ATYTCGGCTTTGAGGGGGCCTG-BBQ	
Influenza B	*M*	InfB-F	GAGACACAATTGCCTACCTGCTT	[26]
		InfB-R	TTCTTTCCCACCGAACCAAC	
		InfB-P	Cy5-AGAAGATGGAGAAGGCAAAGCAGAACTAGC-BBQ	
RSV*	*N*	RSV-F	CTGTCATCCAGCAAATACAC	This study Provided by Taiwan CDC
		RSV-R	GCATATAACATACCTATTAAYCC	
		RSV-P	LC610-ACAGGAGATARTATTGAYACTCCYAAT-BBQ	

* SARS-CoV-2: severe acute respiratory syndrome-coronavirus 2; RSV: respiratory syncytial virus.

The BD MAX system measures these signals at the end of each amplification cycle in real time, and interprets the data to provide a qualitative result for each of the above targets. A positive result for the presence of RNA from one or more of the targets is indicated by the presence of a real-time PCR growth curve and an associated cycle threshold (Ct) value. The BD MAX system software automatically interprets the test result to provide a qualitative result for each of the above targets.

### Analytical tests

The limit of detection (LoD) values for SARS-CoV-2, influenza A/B, and RSV were determined using the AMPLIRUN SARS-CoV-2, INFLUENZA A H1/H3/H1N1, INFLUENZA B, and RESPIRATORY SYNCYTIAL VIRUS (subtype A/B) RNA control (Vircell, Granada, Spain) that contained the purified RNA of the above viral genomes for absolute quantification. All of the above controls were used to prepare a serial dilution panel with the number of replicates ranging from 5 to 10. The analytical sensitivity of the BD MAX platforms was defined as the lowest dilution at which all replicates were identified as positive for SARS-CoV-2, influenza A/B, and RSV.

### Evaluation of speciﬁcity

The speciﬁcity of the lab-developed dual multiplex PCR test was assessed on the BD MAX System against other common upper respiratory viruses (rhinovirus, parainfluenza 1 virus, parainfluenza 2 virus, parainfluenza 3 virus, and adenovirus). These positive samples were obtained from viral cultures of the Taiwan CDC viral infection contract laboratory.

### Clinical performance comparison using clinical samples

We included 205 nasopharyngeal swab specimens from patients hospitalized in the Tri-Service General Hospital, Taipei City, Taiwan. Clinical testing was performed on 205 retrospective clinical specimens, and the results were compared to those obtained with the reference Taiwan CDC recommended methods.

## Results

### Turnaround time for detecting SARS-CoV-2 and other respiratory pathogens on the BD MAX system

We evaluated the total turnaround time per specimen, including sample preparation, RNA extraction, RT-PCR, and interpretation of results, which was compared to that of our current lab-developed PCR method. Using the BD MAX System, with 24 samples processed simultaneously, the turnaround time improved from 4.5 h to approximately 2.5 h, along with a decrease in the hands-on time that in turn reduced the risk of exposure, especially regarding the particularity of the COVID-19 pandemic. Use of multiplex RT-PCR on the BD MAX system shortened the turnaround time by approximately 45% compared the traditional lab-developed method. In addition, we used the test results to identify the RNA of SARS-CoV-2, influenza A/B, and RSV per specimen in a single cartridge.

### Analytic sensitivity of the BD MAX assay detecting SARS-CoV-2, influenza A/B, and RSV

The LoD of the BD Max assay from 10 replicate tests was 9.4 copies per reaction for the *E* and *Orf1ab* genes of RSV (Table 2). We determined the empirical sensitivity of the LoD by preparing serial dilutions using a known concentration of the RNA controls. We accordingly defined the LoD as the minimum concentration with a positive rate of detection of 100%. The LoD for each target was determined to be 50 copies/PCR for the *N1* and *N3* genes of SARS-CoV-2, 200 copies/PCR for the *M* gene of influenza A H1, and 100 copies/PCR for the *M* gene of influenza A H3/H1N1 and influenza B, and for the *N* gene of RSV (Table 2). No cross-contamination was observed when alternating highly positive and negative samples in the same run since separate cartilages were used for each sample.

**Table 2.** Limit-of-detection results for SARS-CoV-2*, influenza A/B, and RSV* on the BD MAX System.

		No. of replicates detected at each dilution/total no. of replicates at indicated no. of copies per PCR						
		1600	800	400	200	100	50	25
	Gene							
SARS-CoV-2	*N1*	5/5 (100)	5/5 (100)	5/5 (100)	10/10 (100)	10/10 (100)	10/10 (100)	2/10 (20)
	*N3*	5/5 (100)	5/5 (100)	5/5 (100)	10/10 (100)	10/10 (100)	10/10 (100)	3/10 (30)
Influenza A H1	*M*	5/5 (100)	5/5 (100)	5/5 (100)	10/10 (100)	4/10 (40)	0/10 (0)	-
Influenza A H3	* *	5/5 (100)	5/5 (100)	5/5 (100)	10/10 (100)	10/10 (100)	4/10 (40)	-
Influenza A H1N1	* *	5/5 (100)	5/5 (100)	5/5 (100)	10/10 (100)	10/10 (100)	4/10 (40)	-
Influenza B	*M*	5/5 (100)	5/5 (100)	5/5 (100)	10/10 (100)	10/10 (100)	6/10 (60)	-
RSV A subtype	*N*	5/5 (100)	5/5 (100)	5/5 (100)	10/10 (100)	10/10 (100)	6/10 (60)	-
RSV^#^ B subtype		5/5 (100)	5/5 (100)	5/5 (100)	10/10 (100)	10/10 (100)	4/10 (40)	-

*RSV, respiratory syncytial virus; SARS-CoV-2, severe acute respiratory syndrome coronavirus 2.

### Analytical specificity of the dual multiplex PCR BD MAX assay

We used samples of known upper respiratory viruses, including rhinovirus, parainfluenza virus, and adenovirus, to evaluate the analytical specificity of the lab-developed dual multiplex PCR test performed on the BD MAX system. Additional undiluted cell culture supernatants were also tested. All test results were found to be highly specific for our intended targets, with no cross-reactivity observed with other upper respiratory viruses (Supplementary Table 1).

### Clinical validation of the BD max assay

A total of 205 clinical samples were included in this study. In particular, using real-time RT–PCR with the lab-developed PCR assay and the BD Max system, we detected 55 samples as positive and 150 samples as negative for SARS-CoV-2. These 55 positive samples were further confirmed in the Taiwan CDC central laboratory. Concordant results were obtained for both assays regarding the detection of SARS-CoV-2, showing 100% agreement (Table 3). Both conventional Taiwan CDC protocols and our dual multiplex PCR on the BD MAX system showed 100% positive agreement for medium and high SARS-CoV-2 viral concentrations (deﬁned as a Ct value < 30.) However, for positive detection cases with Ct values > 30, the BD MAX system showed slightly higher Ct values for the *N1* and *N3* genes compared with the lab-developed method for detection of the *E*-gene, but this did not affect the overall qualitative interpretation of results.

**Table 3.** Positive and negative agreement of the Taiwan CDC lab-developed SARS-CoV-2 assay with the BD MAX platform SARS-CoV-2 assay.

		Lab-developed assay		BD MAX platform	
		*E* gene	*RdRp* gene	*N1* gene	*N3* gene
Total Positives*		55		55	
Ct Value	Low (> 30)	3	11	10	14
	Medium (20–30)	45	41	44	40
	High (< 20)	7	3	1	1
Total Negative^#^		150		150	

* Positive result interpretation: Both *E* and *RdRp* genes were detected or both *N1* and *N3* genes were detected.

^#^ Negative result interpretation: none of *E* or *RdRp* genes was detected or none of *N1* or *N3* genes was detected.

### Rates of coinfection between SARS-CoV-2 and other respiratory pathogens

We retrospectively studied the 205 specimens tested for SARS-CoV-2 and other respiratory pathogens using our dual multiplex RT-PCR on the BD MAX platform. Fifty-five of the 205 specimens (26.8%) were demonstrated to be positive for SARS-CoV-2, whereas none was found to be positive for one or more non–SARS-CoV-2 pathogens compared with the 150 specimens that were negative for SARS-CoV-2. Among the tested specimens, influenza A was the most commonly detected pathogen (n = 19), followed by influenza B (n = 5) and RSV (n = 2) (Table 4). This finding highlighted the importance of differentiating other causes of respiratory illness from SARS-CoV-2, especially during influenza season, because common clinical manifestations of COVID-19, including fever, cough, and dyspnoea, mimic those of other upper-respiratory infections.

**Table 4.** Proportions of specimens positive for non–SARS-CoV-2* respiratory pathogens.

	SARS-CoV-2 status	
	Negative (n = 150)	Positive (n = 55)
	Proportion positive for other respiratory pathogens, n (%)	
Pathogen		
Influenza A	19/150 (12.7%)	0/55 (0%)
Influenza B	5/150 (3.3%)	0/55 (0%)
RSV*	2/150 (1.3%)	0/55 (0%)

*RSV, respiratory syncytial virus; SARS-CoV-2, severe acute respiratory syndrome coronavirus 2.

## Discussion

Taiwan experienced two waves of imported cases of COVID-19: first from China in January to late February, and then from other countries starting in early March. As of 26 November 2020, the self-governing island reported 623 COVID-19 cases, a number that has remained relatively low due to a series of aggressive containment strategies, including mandatory social distancing and wearing masks, quarantine, and monitoring measures that have limited the local transmission of the SARS-CoV-2.

The performance of the Molecular BD Max system for the detection of different pathogens has been evaluated previously [15,27,28]. These studies demonstrated that the BD MAX system is a good diagnostic tool with a rapid turnaround time, enabling appropriate treatment decisions. The BD MAX system offers clinicians a broad suite of clinically relevant and differentiated assays capable of running both Food and Drug Administration-cleared and open system assays. The BD MAX system has been adopted by many medical centres for molecular diagnostics in Taiwan and is distributed globally. However, to our knowledge, this is the first study to develop a lab-based assay for the simultaneous detection of SARS-CoV-2, influenza A/B, and RSV using the BD MAX system. Compared with the in-house results and those from the reference laboratory, the dual multiplex PCR assay on the BD MAX platform developed herein showed good performance. In addition, we validated a rapid and high-throughput method on the BD MAX platform for the accurate and reproducible identification of common upper respiratory tract pathogens, including SARS-CoV-2, avoiding possible contamination and accomplishing fewer hand-on preparation steps.

To date, only three studies have reported the use of multiplex PCR for the detection of SARS-CoV-2 and other common pathogens [29–31]. These two commercial kits, BioFire RP2.1 (BioFire Diagnostics) and QIAstat-SARS (QIAGEN Diagnostics GmbH), have been demonstrated to provide more than 20 detection results, including SARS-CoV-2. However, compared with our BD MAX platform, the single-use cost is much higher for these kits, and their use requires equipping the laboratory with the corresponding instruments; furthermore, only a single specimen can be loaded at a time (Supplementary Table 2).

In summary, our platform is high-throughput, inexpensive, and easier to popularize in several diagnostic laboratories. Further, multiplex assays have been associated with higher reagent costs and lower sample processing capacity per day. However, through this lab-developed PCR method, using the open mode of the BD MAX system, its high-throughput capacity results in lowering the cost and also shortening the turnaround time to 2.5 h by running 24 samples per batch and 192–216 samples in 11 h (depending on the number of batches) per day.

Our developed dual multiplex PCR panel could expand the number of laboratories able to test for SARS-CoV-2 without ignoring coinfecting pathogens as alternative diagnoses for the decided treatment regimen. Using our developed dual multiplex PCR that targets all relevant respiratory viruses in the same cartridge would allow for the detection of other viral infections in patients suspected of having COVID-19. These results suggest that routine testing for non–SARS-CoV-2 respiratory pathogens during the COVID-19 pandemic is important, and might influence disease treatment and clinical decisions. With the advent of the autumn and winter seasons – considered as flu seasons – the second wave of the COVID-19 epidemic might occur at any time. The ability to simultaneously test for influenza A/B, RSV, and SARS-CoV-2 on the BD MAX system would serve as an important infection control management tool in combating the COVID-19 outbreak, and would greatly benefit reference diagnostics laboratories and national governments.

## Supplementary Material

Supplementary_Table_1_and_2_2020_12_11.docx
