# Supplementary material for: Novel dual multiplex real-time RT-PCR assays for the rapid detection of SARS-CoV-2, influenza A/B, and respiratory syncytial virus using the BD MAX open system
Source: Emerg Microbes Infect. 2021 Jan 19;10(1):161–6. doi: 10.1080/22221751.2021.1873073 (PMC7832498; doi:10.1080/22221751.2021.1873073)
Supplement: Supplementary_Table_1_and_2_2020_12_11.docx [file TEMI_A_1873073_SM0894.docx]

**Supplementary Table 1. Analytical specificity of the dual multiplex PCR BD MAX assay**

|  | **Dual multiplex PCR on the BD Max system** | | | | |
| --- | --- | --- | --- | --- | --- |
|  | **SARS-CoV-2** | | **Influenza A** | **Influenza B** | **Respiratory syncytial virus** |
| **Clinical viral isolated with known viruses** | ***N1* gene** | ***N3* gene** | ***M* gene** | ***M* gene** | ***N* gene** |
| Rhinovirus | N.D. | N.D. | N.D. | N.D. | N.D. |
| Parainfluenza 1 virus | N.D. | N.D. | N.D. | N.D. | N.D. |
| Parainfluenza 2 virus | N.D. | N.D. | N.D. | N.D. | N.D. |
| Parainfluenza 3 virus | N.D. | N.D. | N.D. | N.D. | N.D. |
| Adenovirus | N.D. | N.D. | N.D. | N.D. | N.D. |

**^#^N.D., Not Detected**

**Supplementary Table 2.** Comparison of sample-to-results characteristics of three systems.

|  | BioFire RP2.1 | BD MAX System | QIAstat-SARS |
| --- | --- | --- | --- |
| Total turnaround time for 1–24 samples | <1 h* | 2 h 30 min* | <1 h* |
| Reagent cost per sample | 135–150 USD | 9–10 USD | 50–75 USD |
| Maximum throughput per run | 1 sample | 1–24 samples | 1 sample |
| Flexibility | IVD^#^ only | IVD^#^/RUO | IVD^#^/RUO^#^ |
| Ease of use | easy | easy | easy |
| Target pathogen | SARS-CoV-2, influenza A/B, respiratory syncytial virus and other pathogens. | SARS-CoV-2, influenza A/B, and respiratory syncytial virus | SARS-CoV-2, influenza A/B, respiratory syncytial virus and other pathogens. |
| Result interpretation | Programmable interpretation. Ct value and Tm intervals. | Programmable interpretation. Ct value. | Programmable interpretation. Ct value. |
| Onboard reagent traceability | Extraction cassette | Sample Buffer Tube  Extraction cassette | Extraction cassette |

*For BioFire RP2.1 and QIAstat-SARS one sample one time; for BD MAX system, 24 samples at one time

^#^ IVD, in vitro diagnostic devices; RUO, research use only
